# 254. Clinical significance of antineutrophil cytoplasmic antibody positivity in patients infected with SARS-CoV-2

**DOI:** 10.1093/ofid/ofac492.332

**Published:** 2022-12-15

**Authors:** Lucy Eunju Lee, Wooyong Jeong, Yong-Beom Park, Su Jin Jeong, Sang-Won Lee

**Affiliations:** Dongguk University Ilsan Hospital, Seoul, Seoul-t'ukpyolsi, Republic of Korea; National Health Insurance Service Ilsan Hospital, Seoul, Seoul-t'ukpyolsi, Republic of Korea; Yonsei University College of Medicine, Seoul, Seoul-t'ukpyolsi, Republic of Korea; Yonsei University College of Medicine, Seoul, Seoul-t'ukpyolsi, Republic of Korea; Yonsei University College of Medicine, Seoul, Seoul-t'ukpyolsi, Republic of Korea

## Abstract

**Background:**

The Coronavirus Disease 2019 (COVID-19) is well-known for its broad spectrum of immune-related phenotypes similar to those seen in autoimmune or inflammatory diseases. Furthermore, evidence has gradually accumulated that COVID-19 may induce systemic inflammatory manifestations such as multisystem inflammatory syndrome, haemophagocytic syndromes, and systemic vasculitis. Antineutrophil cytoplasmic antibody (ANCA)-associated vasculitis (AAV) is a small vessel vasculitis characterised by necrotising vasculitis. So far, there have been several case reports regarding AAV occurrence after severe acute respiratory syndrome coronavirus 2 (SARS-CoV-2) infection, which have indicated a triggering potential of SARS-CoV-2 infection for AAV occurrence. This study investigated the rate of ANCA positivity and its clinical significance in COVID-19 patients.

**Methods:**

This study included 178 patients infected with SARS-CoV-2 who were enrolled in a cohort of a single center. Myeloperoxidase (MPO)-ANCA and proteinase 3 (PR3)-ANCA from the stored blood sera were measured using the immunoassay kits. Mortality, mechanical ventilator care, and severe infection were assessed as poor outcomes. Severe infection was defined as a medical condition that required a high-flow nasal cannula and/or mechanical ventilator care. The 2022 American College of Rheumatology and the European Alliance of Associations for Rheumatology classification criteria for the three subtypes of AAV were applied only to patients who had MPO-ANCA or PR3-ANCA among the study subjects

**Results:**

The detection rate of ANCA positivity was 18.5%: MPO-ANCA and PR3-ANCA were found in 22 (12.4%) and 14 (7.9%) patients. Patients with ANCA positivity exhibited a lower cumulative survival rate than those without, but the difference was not statistically significant (P = 0.057). However, neither MPO-ANCA nor PR3-ANCA affected the three poor outcomes. According to the new criteria, 12 (6.7%) and 21 (11.8%) patients were classified as having granulomatosis with polyangiitis (GPA) and microscopic polyangiitis (MPA)

**Figure:**
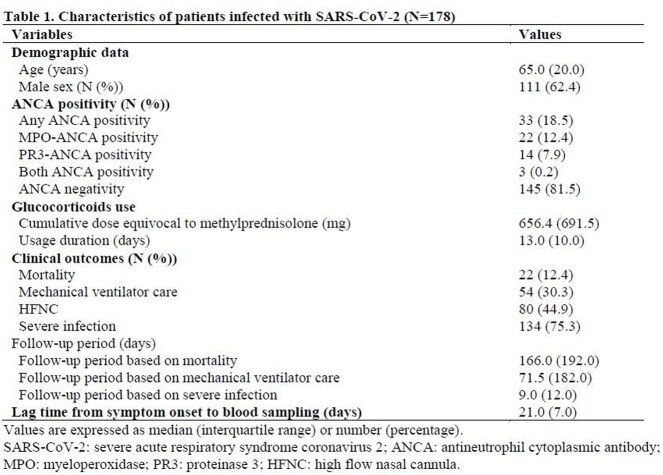

**Figure:**
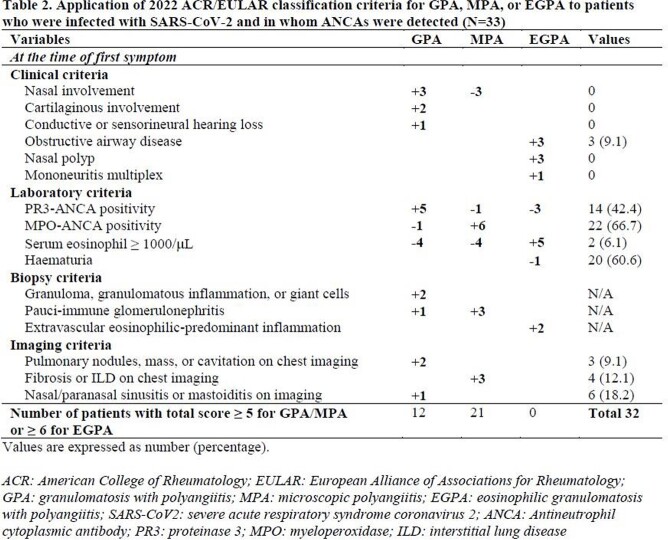

**FIGURE 1.**
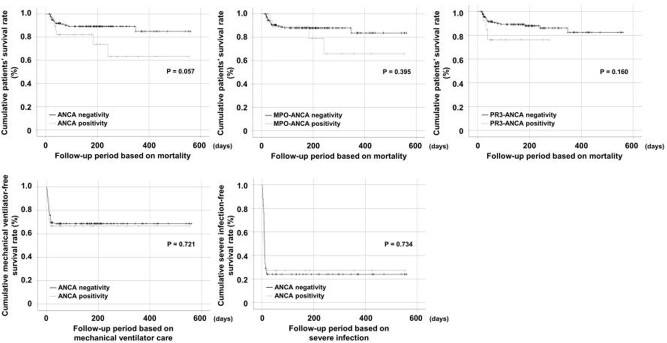
Comparison of the cumulative survival rate

Neither ANCA positivity nor ANCA subtype (MPO-ANCA and PR3-ANCA) positivity had a significant influence on poor outcomes of SARS-CoV-2.

ANCA: antineutrophil cytoplasmic antibody; MPO: myeloperoxidase; PR3: proteinase 3; SARS-CoV-2: severe acute respiratory syndrome coronavirus 2.

**Conclusion:**

SARS-CoV-2 infection may increase the rate of ANCA positivity, which may not affect poor outcomes but contribute to the classification of GPA and MPA despite uncertain clinical significance

**Disclosures:**

**All Authors**: No reported disclosures.

